# Ultrasound-Guided Procedures in the Cervical Spine

**DOI:** 10.7759/cureus.20361

**Published:** 2021-12-12

**Authors:** Bernardo Moreno, Jorge Barbosa

**Affiliations:** 1 Physical Medicine and Rehabilitation, Hospital da Senhora da Oliveira, Guimarães, PRT; 2 Physical Medicine and Rehabilitation, Centro Hospitalar de Lisboa Ocidental, Lisbon, PRT

**Keywords:** medial branch nerve block, cervical facet intraarticular injections, cervical selective nerve root block, cervical spine injections, pain management, ultrasound-guided

## Abstract

Cervical pain is a common symptom among the general population. When conservative strategies fail to provide pain relief, cervical spine injections may be considered. Compared with cervical surgery, cervical injections have low major complications and, with the right indication, have demonstrated good results.

Traditionally, these types of procedures have been performed under fluoroscopy; however, in recent years, ultrasound has become a more common imaging modality to guide spinal injections.

Although ultrasound presents an excellent quality image for soft tissue and allows ​the observation of vascular tissues, nerves, and the contour of bone surfaces, the cervical region has a complicated neurovascular network and a comprehensive understanding of the cervical sonoanatomy should remain as the basis before one can plan cervical ultrasound-guided intervention.

This paper aims to show the advantages of ultrasound in facilitating the performance of cervical spine procedures, including facet joint injections, medial branch blocks, and selective nerve root blocks; analyze the sonoanatomy and landmarks of commonly intervened cervical structures; and illustrate how these procedures can be performed safely and precisely under ultrasound guidance.

## Introduction and background

Cervical spine disorders and cervical pain affect approximately 40% of the population at some time of their lives, increasing its prevalence with advancing age. Most cervical pains have mechanical etiology and improve significantly in about 70% of cases after four weeks, even without specific treatment. However, about 5%-20% of these patients maintain symptoms for more than six months after the onset of pain, characterized by frequent exacerbations with worsening of the pain. When conservative strategies like analgesics or physiotherapy fail to provide pain relief, cervical spine interventions may be considered. Compared with cervical surgery, the cervical injection has low major complications, which makes it more attractive than cervical surgery, which is usually left for patients with neurologic deficits or myelopathic findings [1].

Traditionally, cervical spine injections have been performed under anatomic references or more often with fluoroscopy or computed tomography (CT) guidance. However, in recent years, there has been a big rise in the use of ultrasound (US) in pain medicine, including with cervical pathology, as evidenced by the remarkable increase in the scientific literature about US-guided injections in the management of chronic neck pain [2].

The purpose of this article is to analyze the sonoanatomy of the commonly intervened cervical structures and to illustrate how those procedures can safely and precisely be performed under US guidance.

Methods

We performed a literature search of the MEDLINE database from January 2010 to December 2020 using the search terms “ultrasound-guided,” “pain management,” and “cervical spine injections” and different selected nerves or structures relevant in this review such as “cervical selective nerve root block,” “cervical facet intraarticular injections,” and “medial branch nerve block.”

## Review

Ultrasound in the cervical intervention

Besides being easily available, portable, low cost, radiation-free, and with no known contraindications, US is a unique imaging method as it uses ultrasonic waves to create dynamic, high-resolution images in real time, presenting excellent quality images for soft tissue. It also allows observing vascular tissues, nerves, and the contour of bone surfaces, as well as needles and different injectable products during administration. Because of these characteristics, US is particularly beneficial for cervical spine injections, where a multitude of vulnerable vessels, nerves, and other vital soft-tissue structures are confined to a small area and are often in the path of the needle trajectory [3]. In addition, US-guided procedures have a shorter duration compared to those guided by fluoroscopy and do not result in contrast medium-related allergic reactions [4].

Despite these advantages, US has some limitations in neuraxial (epidural or intrathecal) procedures as it has a limited resolution at deep levels and near bony surfaces that affect image quality, not been possible to visualize the real-time propagation of the injectable in the epidural or intrathecal space. In addition, cervical sonoanatomy is actually quite complicated and US experience combined with good cervical anatomy knowledge is needed to perform US-guided injections on the cervical region [5].

Cervical medial branch and facet joint injections

The prevalence of cervical facet joint degeneration arthropathy in chronic neck pain is reported to be 35%-55% [6]. Clinical and radiological findings together are usually needed to attribute neck pain to facet joints. Intra-articular facet joint injections have been used for diagnosis confirmation and facet joint-related pain treatment, although there is a lack of evidence as regards effective relief following these injections. Therefore, cervical medial branch block is still proposed as the standard diagnostic method for diagnosing/treating facet joint-related neck pain [7].

Injection or radiofrequency neurotomy of the cervical medial branch (innervating one or two consecutive facet joints) is reported as a reliable method for diagnostic confirmation and treatment of chronic cervical facet joint-related pain [8]. Since a single block might yield a false positive response (38%), a second confirmatory injection on a different day is required if radiofrequency neurotomy of the medial branch is planned. Third occipital nerve (TON) neurotomy is reported as an effective treatment method for headaches associated with the TON and C2-C3 facet joint-related pain [9].

The great occipital nerve (GON) is the primary sensory nerve of the occipital area. Its blockade is indicated in the case of GON entrapment underneath the obliquus capitis inferior muscle, a disorder frequently referred to as occipital neuralgia [10], and has been shown to be effective in various headache syndromes, such as primary headache, tension headache, cluster headache, migraine, and cervicogenic headache [11].

Anatomy

Cervical zygapophyseal (facet) joints are diarthrodial joints, containing both fibrous joint capsule and synovial membrane [2]. Cervical vertebrae share common characteristics at C3 to C6 levels while C1 (atlas), C2 (axis), and C7 vertebrae have unique anatomic features [12]. While transverse processes of the C3-C6 vertebra have anterior and posterior tubercles, the C7 transverse process has only a posterior tubercle with a missing anterior tubercle [13].

The C1 vertebra does not have a vertebral body or spinous process but has the longest transverse process among the cervical spine. The C2 vertebra has a prominent bifid spinous process, and dens projecting superiorly to form a central joint with the C1 vertebra, while the C7 vertebra has the longest spinous process among all cervical vertebrae [10]. Each facet joint is formed by the superior articular process (SAP) of one cervical vertebra articulating with the inferior articular process (IAP) of the vertebra above, at the level of the junction of the lamina with the pedicle. The angulation of the facet joint increases caudally, being about 45º superior to the transverse plane at the upper cervical level to assume a more vertical position at the upper thoracic level. The SAP also faces more posteromedially at the upper cervical level, changing to more posterolaterally at the lower cervical level, with C6 being the most common transition level [2].

Cervical zygapophyseal joints are innervated by articular branches derived from cervical medial branches and contain free and encapsulated nerve endings, nociceptors, and mechanoreceptors. For medial branches of C4 and cervical caudal levels, they arise from the dorsal rami of the respective cervical spinal nerves, passing over the base of their corresponding transverse process and coursing posteriorly to encircle through the center of the corresponding articular pillars. Here, cervical medial branches have a constant relationship with the bone at the dorsolateral aspect of the articular pillar, as they are bound to the periosteum by an investing fascia and held in place by the tendon of the semispinalis capitis muscle. Then articular branches arise as the nerve approaches the articular pillar posterior localization. One articular branch for the innervation of the superior zygapophyseal joint and another articular branch for the innervation of the inferior facet joint, so each cervical facet joint has double innervation, one from the medial branch above and the other from the medial branch below its position [2]. Different from the other roots, the dorsal ramus of the C3 spinal nerve has a superficial and a deep medial branch. The deep medial branch circulates the C3 articular pillar, in the same way as the other cervical medial branches and innervates the C3-C4 zygapophyseal joint, while the superficial medial branch of the C3 dorsal ramus is the largest cervical medial branch and is known as the TON [10]. It lies around the lateral and posterior side of the C2-C3 facet joint, supplying articular branches to the joint. Herewith, unlike the C3-C4 facet joint below which they have dual innervation from two consecutive medial branches, the C2-C3 facet joint is innervated by the TON [1]. Consequently, blocking the ipsilateral TON as it passes the lateral aspect of the joint can be done to treat pain derived from the C2-C3 zygapophyseal joint and the block of cervical medial branches as they cross the waists of the superior and inferior articular pillars of the corresponding joint can be done to treat pain derived from the joints below C2-C3 [2].

The GON is the medial branch of the dorsal ramus of C2. This nerve arises between C1 and C2 vertebrae and, after emerging from the suboccipital triangle, ascends beneath the obliquus capitis inferior muscle. Here, it goes between the obliquus capitis inferior and the semispinalis capitis muscles and then passes through the tendinous aponeurosis of the trapezius muscle on the nuchal line and ascends to innervate the posterior neck and the vertex of the occiput skin. The GON can be entrapped at multiple points, particularly between the obliquus capitis inferior and the semispinalis capitis muscles [1].

Evidence of US-Guided Cervical Zygapophyseal Joints Intra-articular Procedures

Jochen Obernauer et al. concluded that the US-guided facet joint injection in the middle and lower cervical spine is accurate, feasible, and bears the minimal risk. It results in a significant pain reduction, not different from CT-guided instillations. Additionally, a reduction of time, radiation dose, and resources are highly evident [14]. Galiano et al. studied the viability of US as a guide for cervical facet joints intra-articular injections in cadavers. With a lateral approach, it was possible to identify the C2-C3 to C6-C7 facet joints in 36 of 40 attempts. CT was used to verify the location of the needle tips within the joint space. In this context, the use of US guidance has been advocated for performing facet joint injections [2].

Evidence of US-Guided Cervical Medial Branch Block

In a study, Eichenberger et al. divulged the use of US guidance in the TON block. The needles were positioned with US guidance and then verified by fluoroscopy. In all participants, it was possible to observe the TON and the C2-C3 zygapophyseal joint was identified correctly by US in 27 of 28 cases. A total of 23 needles were correctly situated into the target area and it reported the feasibility of needle position in 82% of injections with a 90% success of nerve block, confirmed by fluoroscopy [2].

Sonoanatomy and US-Guided Technique for Cervical Zygapophyseal ​​​​Joints Intra-articular Injections

Lateral approach: With the patient lying in the lateral decubitus position, a US examination of the cervical spine is performed using a high-resolution linear-array transducer. The transducer is applied transversely to the lateral aspect of the neck to obtain a short-axis view of the cervical spine. One can easily identify the cervical transverse process with the anterior and posterior tubercles as hyperechoic structures presenting the “2-humped camel” sign, forming a wall and the thin lamina on its floor. The nerve root can be identified as a round or oval hypoechoic structure between the anterior and posterior tubercles.

**Figure 1:**
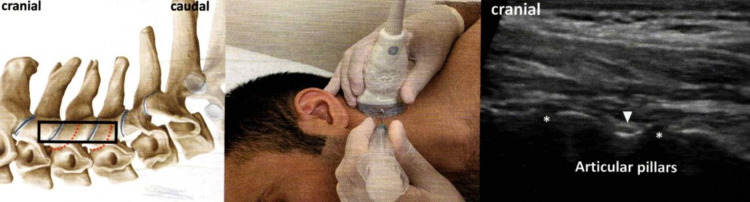
Cervical facet joints injections - lateral approach. The probe (black rectangle) is positioned in a longitudinal plane over the posterolateral neck (left image). In this position, it is possible to target the cervical facet joints using the out-of-plane, anterior to posterior approach, while the patient is in the lateral decubitus position (central image). The corresponding US scan (right image) shows the facet joints (white asterisks) and the medial branches (white arrowhead) as hypoechoic dots located in the grooves of the articular pillars. Originally published in Akkaya N, Constantino J: Upper Cervical Injections. Ultrasound Imaging & Guidance for Musculoskeletal Interventions in Physical and Rehabilitation Medicine. Özçakar L (ed): Edi-ermes, Milan, Italy; 2020. 35-47 [1]. Used with copyright permission from the original publisher.

The cervical level can be recognized by the identification of the transverse process of the C7 and C6 vertebrae. The C7 transverse process, unlike the superior levels, has only a prominent posterior tubercle. At this point, by moving the probe cranially, the C6 transverse process appears with the characteristic sharp anterior tubercle (also named Chassaignac tubercle), which is longer than the posterior tubercle, and thereafter, the consecutive upper cervical spinal levels can be easily identified. At the C6 foramen level, the C5-C6 facet is visualized [15]. After the desirable cervical level is identified, by moving the transducer posteriorly, in a short-axis view, the superior articular and the inferior articular processes forming the facet joint come into the image as hyperechoic signals with the joint space in the middle as an anechoic gap. The needle can be introduced lateral to the probe and moved from posterior to anterior to the target (joint space) in an in-plane approach [12]. This view offers better visualization of blood vessels in the vicinity of the injection zone. Alternatively, the probe can be placed in a long-axis view of the cervical spine at the targeted level and the needle can be inserted into the joint space, on the “hill,” through an out-of-plane approach, from anterior to posterior (Figure 1) [1].

Posterior approach: Reviewing the literature, several authors prefer this approach for different motives. Bilateral injections can be performed without the need to change the patient's position and it is easier to identify the desirable cervical level with the patient in the prone position [1,2].

Posterior short-axis spine view: A curved or a linear probe can be used, depending on the patient's neck size. Bony landmarks are occiput and the spinous processes of cervical vertebrae and the counting of the cervical spinous process can be used for achieving the desirable cervical level. At upper cervical levels, one can start counting from cranial to caudal with the probe in a short-axis view of the cervical spine at the midline (cervical spinous processes). The C1 vertebra has no or has only an elementary spinous process and the first spinous process observed is a bifid spinous representing the C2 level. If it is more adequate to start counting from caudal to cranial, the C7 spinous process can be visualized for being the most prominent cervical spinous process. After the respective cervical segment is defined, the transducer is centered over the spinous process. By moving laterally and slightly tilting and pivoting the transducer in this axial position, the respective facet joint becomes visible. Subsequently, a needle can be inserted from a dorsomedial approach in an in-plane technique, which enables visualization of the complete needle path in real time [1,2].

Posterior long-axis spine view: This procedure can also be made in a long-axis spine view. Starting at the initial position with a short-axis view, at the desirable spinous process level, by moving the transducer into a long-axis view and laterally, it is possible to see the lamina. More laterally, the facet column will come into the image as a characteristic “saw sign.” The IAP of the level above and the SAP of the level below appear as hyperechoic lines, and the joint space appears as an anechoic cleft in between.

**Figure 2:**
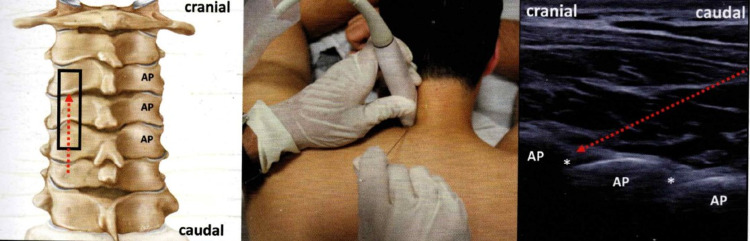
Cervical facet joints injections - posterior long-axis spine approach. In the left image, the probe (black rectangle) is positioned in a longitudinal plane (left image), making it possible to inject the cervical facet joint(s) using an in-plane, caudal to cranial approach (central image). The corresponding US scan (right image) shows the articular processes (AP) as hyperechoic lines and the joint spaces as anechoic clefts (white asterisks) in between. Red dotted arrow: needle's pathway. Originally published in Akkaya N, Constantino J: Upper Cervical Injections. Ultrasound Imaging & Guidance for Musculoskeletal Interventions in Physical and Rehabilitation Medicine. Özçakar L (ed): Edi-ermes, Milan, Italy; 2020. 35-47 [1]. Used with copyright permission from the original publisher.

The needle is inserted inferior to the caudal end of the transducer and advanced from distal to proximal, in an in-plane approach, to enter the inferior part of the joint (Figure 2) [1].

Sonoanatomy and US-Guided Technique for Cervical Medial Branch Block

For a TON block, the patient is placed in lateral decubitus position and a high-frequency linear transducer is placed in a coronal plane with the cephalic end of the transducer on the mastoid process. The probe is slid caudally to find the C1 transverse process and then about 1-2 cm more caudally until finding the transverse process of C2. Next, the transducer is moved 5-8 mm posteriorly until the C2 articular pillar has been well visualized. The transducer is then translated caudally until the visualization of the C2-C3 joint along with the TON appearing as a hypoechoic oval structure in the apex of the C2-C3 facet joint hypoechoic gap.

To visualize the deep C3 and the C4-C6 medial branches, once the C2-C3 facet joint is identified, maintaining a coronal view, the probe is carefully moved in a caudal direction to observe the lower cervical zygapophyseal joints until the desired level is achieved [15]. The deep C3 medial branch is a hypoechoic oval structure seen in the short axis in the waist of the C3 articular pillar, between the C2-C3 and C3-C4 facet joints, and the same applies to C4-C6 medial branches, located in the deepest point between two consecutive facet joints, in contrast to TON.

Alternatively, also with the patient in lateral decubitus position and with the probe in an axial view, it is possible to locate the C7 vertebra for its transverse process with only one (posterior) tubercle, and the C6 transverse process for its anterior and posterior tubercle. The higher cervical levels are identified by moving the transducer cranially. At the desirable level, the probe is shifted into a coronal view and to a slightly posterior position to visualize the facet joint and cervical medial branches.

**Figure 3:**
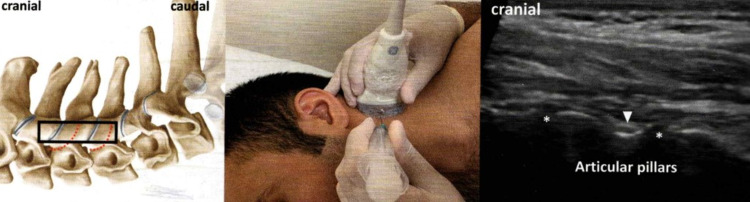
Cervical medial branch injection. The probe (black rectangle) is positioned in a longitudinal plane over the posterolateral neck (left image). In this position, it is possible to target the cervical medial branches (red dotted lines) using the out-of-plane, anterior to posterior approach, while the patient is in the lateral decubitus position (central image). The corresponding US scan (right image) shows the facet joints (white asterisks) and the medial branches (white arrowhead) as hypoechoic dots located in the grooves of the articular pillars. Originally published in Akkaya N, Constantino J: Upper Cervical Injections. Ultrasound Imaging & Guidance for Musculoskeletal Interventions in Physical and Rehabilitation Medicine. Özçakar L (ed): Edi-ermes, Milan, Italy; 2020. 35-47 [1]. Used with copyright permission from the original publisher.

For a medial branch block, with the spine in long-axis, the procedure is similar to the facet joint injection with the needle being advanced from anterior to posterior, in an out-of-plane approach, aiming the bone surface depending on our target. The apex of the convexity of the C2-C3 joint constitutes the target point for the TON block. For C3-C6 medial branches blocks, the target will be the hypoechoic oval structure lying in the middle of hyperechoic “valleys” (Figure 3). If medial branches are not visualized, the “valleys” apex would be the target [2].

Because all vital structures are located anterior to the facet joint, the insertion of the needle in the approach mentioned above reduces the risk of unintentional injury when the needle tip is not precisely visible [1]. Alternatively, the needle can be introduced caudal to the probe and moved under the real-time US to the desirable nerve, in an in-plane view [2].

Unlike the C3-C6 medial branches, the target for the C7 medial branch is located caudal to the C6-C7 facet joint, but proximal to the cranial edge of the C7 transverse process [16].

Different authors believe that even for an experienced sonographer, visualizing very small structures, such as cervical medial branches, may be challenging, especially in obesity and medial branch deep localization cases. So targeting the bony landmarks even without direct visualization of the medial branch might be a good option [8].

For achieving the GON, the patient lies in a prone position or seated with a slight cervical flexion to expose the occiput. The transducer is placed obliquely between the C1 transverse process and the C2 spinous process. In this view, the inferior obliquus capitis and semispinalis capitis muscles can be seen located lateral to the spinous process, while the GON appears as a hypoechoic round or oval structure situated within the plane of these two muscles. Doppler images can be used to visualize any branch of the occipital artery next to the nerve.

For GON block, the needle is inserted from lateral to medial using the in-plane technique and targeting the hypoechoic round structure (GON) between the inferior obliquus capitis and semispinalis capitis muscles. The needle tip should be advanced until being close to the GON sheath [1].

Cervical selective nerve root (transforaminal) block

Cervical radiculopathy is a common condition with an annual incidence of 83 per 100,000. More frequently, it can be caused by borderline lateral and intraforaminal disc herniation or foraminal stenosis. In the last years, with initial conservative treatment failure, physicians have been considering a cervical epidural steroid injection administered in the cervical spine via an interlaminar or a transforaminal route, usually guided by fluoroscopy. In general, cervical epidural steroid injections have been a subject of intense debate as they have been associated with significant problems resulting from vertebral artery injuries and/or brain stem and spinal cord infarctions. Recently, because of its capacity to visualize soft tissue structures, nerves, vessels, and the spread of the injectate, various study groups have shown the reliability of US guidance for injections with a steroid and/or local anesthetic, in patients with cervical radiculopathy, unresponsive to conservative treatments [4]. Although spreading the solution around the cervical nerve root can be monitored by the real-time US, it is difficult to do through the foramen into the epidural space due to the transverse process bony artifact, so therefore this technique has to be referred to as “selective cervical nerve root block” rather than “cervical transforaminal epidural injection” [2].

Anatomy

The brachial plexus is composed of the ventral rami of C5-C8 and T1 nerve roots. It is anatomically divided into five portions: roots, trunks, divisions, cords, and terminal branches. Emerging from the spinal cord, the cervical nerve roots pass anterolaterally through the neural foramen, which is limited anteriorly by the vertebral body and the intervertebral disc, and posteriorly by the articular pillars. The inferior part of the neural foramen is occupied by the cervical spinal nerve roots and the epiradicular veins reside at the foramen superior part [2]. The radicular arteries, supplying the spinal cord, arise from the vertebral, ascending, and deep cervical arteries and they are in proximity to the spinal nerve roots [1]. Hoeft et al., in a cadaver dissection study, demonstrated that radicular artery branches from the vertebral artery cross over the foramen anteromedial side, whereas those that come from the ascending or deep cervical arteries, have the greatest clinical significance because it is suggested that they course medially, crossing the foramen. Variable anastomoses between the vertebral and cervical arteries can exist, making it possible to introduce steroid particles into the vertebral circulation via cervical arteries [2].

Evidence of US-Guided Cervical Nerve Root Block

There have been more and more studies comparing the efficacy and safety of US-guided cervical selective nerve root injections with fluoroscopy-guided cervical epidural steroid injections via an interlaminar or a transforaminal route in patients with cervical radiculopathy.

Jee et al. compared short-term treatment effects between US-guided cervical selective nerve root block and fluoroscopy-guided transforaminal cervical epidural steroid injection and found no significant intergroup differences [4]. In addition, Park et al. found that pain relief and functional improvements were similar with fluoroscopy-guided interlaminar cervical epidural steroid injections and US-guided selective nerve root blocks [4].

Jang et al.'s results suggest that, compared with fluoroscopy-guided transforaminal and interlaminar cervical epidural steroid injection, US-guided selective nerve root block has a low intravascular injection rate, stating that it is unlikely that serious complications could occur. Also, US-guided selective nerve root block requires a shorter procedure time while providing similar pain relief and functional improvements. Therefore, it was suggested that for the treatment of patients with lower cervical radicular pain, US-guided selective nerve root block should be recommended as a first-line method [17].

Sonoanatomy and US-Guided Technique for Cervical Selective Nerve Root Block

**Figure 4:**
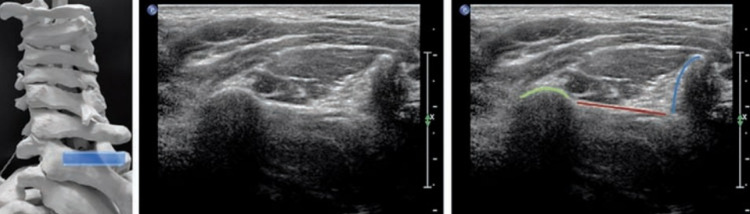
Posterior axial scan plane of the spinous process at C7 level. Posterior axial scan plane of the spinous process (blue) at C7 level. Vertebral arch: red; articular process: light green. Originally published in Loizides A, Obernauer J, Peer S, Bale R, Galiano K, Gruber H: Ultrasound-guided injections in the middle and lower cervical spine. Medical Ultrasonography. 2012, 14:235-8. http://www.medultrason.ro/ultrasound-guided-injections-in-the-middle-and-lower-cervical-spine/ [13]. Used with copyright permission from the original publisher.

**Figure 5:**
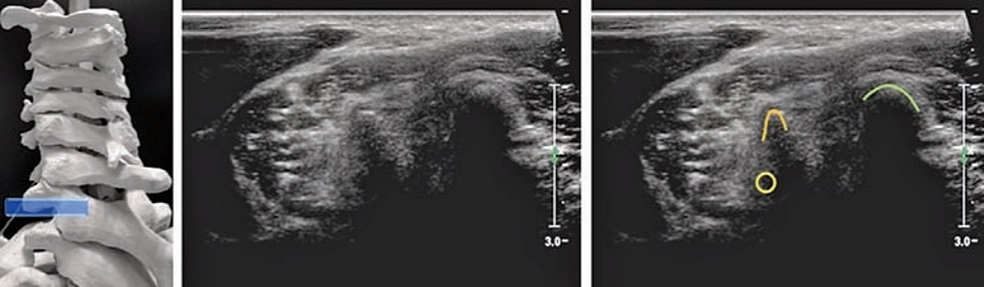
Posterior axial scan plane of the transverse process at the C7 level. Note the missing anterior tubercle. Articular process: light green; posterior tubercle: orange; C7 nerve root: yellow. Originally published in Loizides A, Obernauer J, Peer S, Bale R, Galiano K, Gruber H: Ultrasound-guided injections in the middle and lower cervical spine. Medical Ultrasonography. 2012, 14:235-8. http://www.medultrason.ro/ultrasound-guided-injections-in-the-middle-and-lower-cervical-spine/ [13]. Used with copyright permission from the original publisher.

With the patient lying in lateral decubitus position, US imaging of the cervical spine is performed using a high-resolution linear transducer. In an exact midline axial scan, the spinous processes of the lower cervical spine are defined. From this midline position, the transducer is offset laterally along the vertebral arch and the respective articular process (Figure 4) towards the transverse process. As already mentioned, the cervical level is easily identified after the recognition of the transverse process of the C7 and C6 vertebrae. At the level of the cricoid cartilage, the C6 transverse process comes into the image with its posterior and anterior tubercles (the latter is longer) and below, with its unique characteristic, the C7 transverse process with its only posterior tubercle (Figure 5). While there are seven cervical vertebrae, there are eight cervical nerve roots. From C1 to C7, each nerve root emerges from the intervertebral foramen, as a hypoechoic round-to-oval structure, sliding on the groove ventral to the posterior tubercle of the transverse process of its level [13]. The C8 nerve root emerges between C7 and T1 and is located in the lower portion of the intervertebral foramen, closely related to the periradicular vessels [18].

**Figure 6:**
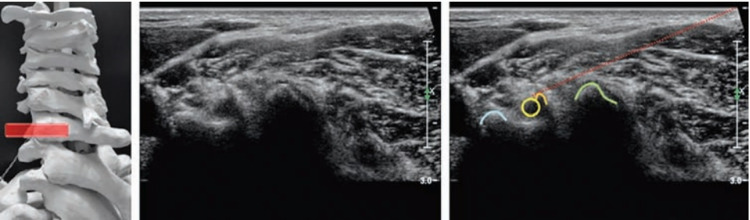
Ultrasonography-guided selective nerve root block (target C6 nerve root). Articular process: light green; posterior tubercle: orange; anterior tubercle: light blue; C6 nerve root: yellow; intended needle path placed on the dorsal surface of the C6 nerve root: red. Originally published in Loizides A, Obernauer J, Peer S, Bale R, Galiano K, Gruber H: Ultrasound-guided injections in the middle and lower cervical spine. Medical Ultrasonography. 2012, 14:235-8. http://www.medultrason.ro/ultrasound-guided-injections-in-the-middle-and-lower-cervical-spine/ [13]. Used with copyright permission from the original publisher.

Once the appropriate spinal level and cervical nerve root are identified with the transducer in short-axis view, through probe manipulation, the best possible image is sought to locate the nerve root and the surrounding vessel [4]. It is suggested to turn on the power/color Doppler mode to confirm the vascular structures localization [1]. The vertebral artery is seen anterior to the nerve root. Next, via an in-plane approach, the needle is inserted from posterior to anterior, toward the corresponding cervical nerve root at the external foraminal opening, between the anterior and posterior tubercles of the transverse process, except for the C7 transverse process, which only has a posterior tubercle. After the needle passes through the posterior tubercle, the needle end is placed on the dorsal side of the nerve (Figure 6). At this location, the injectable product is distributed around the nerve root [7]. Although spreading the solution around the cervical nerve root can be monitored by the real-time US, on certain occasions, it can be difficult because of transverse process bony artifact, so it is important to take special caution to ensure that no intravascular injection is made. In addition to the confirmation of the absence of vascular structures near the needle tip with power/color Doppler mode, prior to injection, it is important to have no abnormal findings with careful aspiration and to use no particulate steroid with local anesthetic as the treatment drug to inject [4].

## Conclusions

Pain intervention under US guidance is particularly valuable in peripheral and musculoskeletal procedures and, over the last years, there has been a growing interest in US as an imaging modality for spinal interventions. It has advantages over CT and fluoroscopic guidance and should be considered as an option among modalities for cervical injections guidance.

The major advantages of US are that it is radiation-free, low cost, has an excellent quality image for soft tissue, and also allows observing vascular tissues, nerves, the contour of bone surfaces, as well as needles and different injectable products during administration, with high-resolution images in real time.

Several anatomic and clinical studies demonstrated that safe cervical procedures can be performed under US guidance with precision and clinical efficacy. Although with these advantages, a comprehensive understanding of the cervical sonoanatomy should remain as a pre-requisite before one can plan US-guided cervical interventions as ultrasonographic scanning of cervical structures is quite difficult compared to extremities scanning.

Precise recognition of the shape of the cervical vertebrae, neck muscles, and cervical neurovascular vital structures is essential for a secure and effective procedure. In addition to good clinical knowledge about correct indications, physicians are suggested to be familiarized with peripheral scanning and injection techniques of the limbs before proceeding to intervention on the cervical region.
